# Channel and Spatial Attention in Chest X-Ray Radiographs: Advancing Person Identification and Verification with Self-Residual Attention Network

**DOI:** 10.3390/diagnostics14232655

**Published:** 2024-11-25

**Authors:** Hazem Farah, Akram Bennour, Neesrin Ali Kurdi, Samir Hammami, Mohammed Al-Sarem

**Affiliations:** 1 Laboratory of Mathematics, Informatics and Systems (LAMIS), Echahid Chiekh Larbi Tebessi University, Tebessa 12002, Algeria; farah.hazem@univ-tebessa.dz; 2 College of Computer Science and Engineering, Taibah University, Medina 41477, Saudi Arabia; nkordi@taibahu.edu.sa (N.A.K.);; 3 Department of Management Information Systems, Dhofar University, Dhofar, Salalah 211, Oman; samir@du.edu.om

**Keywords:** self-residual attention network (SRAN), chest X-ray, biometrics, person identification, person verification

## Abstract

Background/Objectives: In contrast to traditional biometric modalities, such as facial recognition, fingerprints, and iris scans or even DNA, the research orientation towards chest X-ray recognition has been spurred by its remarkable recognition rates. Capturing the intricate anatomical nuances of an individual’s skeletal structure, the ribcage of the chest, lungs, and heart, chest X-rays have emerged as a focal point for identification and verification, especially in the forensic field, even in scenarios where the human body damaged or disfigured. Discriminative feature embedding is essential for large-scale image verification, especially in applying chest X-ray radiographs for identity identification and verification. This study introduced a self-residual attention-based convolutional neural network (SRAN) aimed at effective feature embedding, capturing long-range dependencies and emphasizing critical spatial features in chest X-rays. This method offers a novel approach to person identification and verification through chest X-ray categorization, relevant for biometric applications and patient care, particularly when traditional biometric modalities are ineffective. Method: The SRAN architecture integrated a self-channel and self-spatial attention module to minimize channel redundancy and enhance significant spatial elements. The attention modules worked by dynamically aggregating feature maps across channel and spatial dimensions to enhance feature differentiation. For the network backbone, a self-residual attention block (SRAB) was implemented within a ResNet50 framework, forming a Siamese network trained with triplet loss to improve feature embedding for identity identification and verification. Results: By leveraging the NIH ChestX-ray14 and CheXpert datasets, our method demonstrated notable improvements in accuracy for identity verification and identification based on chest X-ray images. This approach effectively captured the detailed anatomical characteristics of individuals, including skeletal structure, ribcage, lungs, and heart, highlighting chest X-rays as a viable biometric tool even in cases of body damage or disfigurement. Conclusions: The proposed SRAN with self-residual attention provided a promising solution for biometric identification through chest X-ray imaging, showcasing its potential for accurate and reliable identity verification where traditional biometric approaches may fall short, especially in postmortem cases or forensic investigations. This methodology could play a transformative role in both biometric security and healthcare applications, offering a robust alternative modality for identity verification.

## 1. Introduction

In forensic science, biometric modalities play a critical role in identifying and verifying individuals. Old biometrics modalities are often used for identification and verification. The number of homicide cases has increased many times over along with population growth. In forensic, with the rise and ever-increasing potential of deep learning techniques in recent years, there are many modalities that could be used to identify human identity in postmortem cases, such as fingerprints, DNA analysis, facial recognition, iris recognition, odontology (dental records), ear shape recognition, and vein pattern recognition [1,2,3,4,5]. However, each modality also comes with limitations that can affect its reliability and usability in forensic investigations. Traditional biometric modalities in forensic investigations face significant limitations in cases where the human body is severely damaged or has been deceased for a long time, decomposed, or altered by trauma, burns, or prolonged exposure to the elements [6,7]. For example, fingerprint recognition becomes nearly impossible if the skin is burned or decayed, and facial recognition may fail if the face is disfigured [8,9]. DNA analysis can also be hindered by contamination or degradation of biological samples [10]. Additionally, methods such as iris or vein pattern recognition are impractical when eyes or limbs are damaged. These scenarios complicate the identification process and often require alternative forensic methods or the use of multiple biometric modalities to achieve reliable results. While these methods can provide valuable insights, many of them not only are time consuming but face limitations due to the absence of comprehensive data. For instance, DNA recognition, while highly accurate, often involves extensive processing times that can delay investigations. In cases where biological samples are degraded, scarce, or contaminated, the reliability of DNA matching diminishes significantly. In this case, we recommend identifying individuals using chest X-ray images. Chest X-ray identification is particularly useful because unlike in other biometric methods, the skeletal structure remains largely unaffected by time [11,12,13]. Even in cases of advanced decomposition or long periods since death, the bones and specific patterns in the chest remain relatively stable, allowing for identification when other methods, such as fingerprints or facial recognition, are no longer viable [13,14,15]. Studies have shown that radiographs are effective in comparing antemortem and postmortem X-rays for positive identification, with intervals extending several months to years in some forensic cases [16,17]. The skeletal system’s unique features, such as disease-related or postsurgical changes, provide durable, identifiable markers. Key regions, including the clavicles, vertebrae, and skull, offer consistent anatomical markers for comparison over extended periods postmortem [17,18]. Chest radiography remains effective in scenarios involving decomposed remains, burn victims, and submerged bodies, where traditional identification methods may fail [16]. Even in cases of age-related factors or the development of new pathologies in the chest, chest X-rays remain effective for identification tasks [17,18]. In recent years, the development of automated systems using image processing has further streamlined this identification process, increasing speed and consistency [19]. Radiographic identification accuracy has shown results comparable to those of fingerprint analysis (dactyloscopy), maintaining its relevance even with the advent of DNA analysis [18]. Additionally, chest X-rays are often available in medical records, especially for individuals with a history of lung or heart conditions [15]. This existing data can be invaluable in forensic investigations, providing a reliable means of comparison even when other identifying information is unavailable or has been compromised. This availability of historical medical data, combined with the durability of skeletal features, makes chest X-rays a powerful tool in time-sensitive and challenging forensic cases [20]. However, chest X-ray radiographs require extensive work to extract discriminative features. To effectively apply this modality, it is essential to ensure that the maximal intraclass distance is smaller than the minimal interclass distance within a defined metric space. In this paper, we propose a modified backbone of a Siamese neural network leveraging CNNs and a self-residual attention block (SRAB). The SRAB represents a paradigm shift in feature extraction, incorporating both channel and spatial attention mechanisms. These mechanisms enabled the model to discern intricate patterns within radiographs, capturing salient information crucial for accurate identification. The self-residual attention network (SRAN) operated through a unique blend of channel and spatial attention mechanisms, each finely tuned to enhance feature representation within chest X-ray radiographs. The channel attention mechanism selectively amplified important channels by assigning attention weights based on global significance, while the spatial attention mechanism highlighted crucial spatial locations through independent attention weight assignments across channels. These mechanisms collectively refined feature maps to capture intricate patterns and relevant information essential for accurate person identification. By seamlessly integrating SRABs into the ResNet50 backbone, leveraging the pretrained ResNet50 base, the model enhanced its feature learning capabilities, thereby achieving unprecedented levels of accuracy and reliability in person identification and verification tasks. Central to the success of this methodology was the utilization of triplet loss, a powerful optimization technique that facilitated the learning of a discriminative embedding space and best representation to chest features. By minimizing intraclass similarities and maximizing interclass dissimilarities, the model learned to distinguish individuals based on their chest X-ray radiographs with exceptional precision. The clever combination of Siamese architecture and triplet loss within powerful deep learning frameworks, augmented with an attention mechanism to enrich discriminative feature learning, maximized interclass distances and minimized intraclass variations by optimizing Siamese networks for accurate image similarity comparison and improving the embedding space through triplet loss [21].

This paper makes the following contributions.

- Advancements in Biometrics: This work demonstrates the potential of chest X-rays as a reliable biometric modality, enhancing the scope and precision of biometric identification in medical imaging.
- Development of a Biometric System: A novel system is proposed for human identification and verification using chest X-ray radiographs, leveraging distinctive radiographic features.
- Innovative Use of Siamese Networks: A novel approach utilizing Siamese networks is introduced, enabling the learning of highly discriminative features by comparing and contrasting pairs of images.
- Enhanced Discriminative Power with Triplet Loss: The incorporation of triplet loss further improves the model’s discriminative capability, driving the network to learn a feature space that maximizes the dissimilarity between different individuals.
- Establishing a New Benchmark: A new standard is established for person identification in chest X-ray imaging, setting a benchmark for future research in this area.
- Introduction of the Self-Residual Attention Network (SRAN): The paper introduces the SRAN architecture for chest X-ray image analysis, advancing the field with this attention-based approach.

This study embarks on a comprehensive exploration of foundational concepts, delving into existing research to provide a holistic overview in the “Related Works” section. Subsequently, Section 3 elucidates the meticulously devised structure underlying our study, serving as a solid foundation for the experiments detailed in the ensuing section. In Section 4, a thorough analysis unveils valuable insights contributing to the broader discourse in the field. Finally, the Section 5 consolidates the amassed data, underscores its significance, and outlines potential avenues for future research, thus offering valuable recommendations for further exploration.

## 2. Related Works

Through a comprehensive review of the current literature, we identified recent approaches, existing knowledge gaps, and areas in need of further investigation. This section offers an overview of studies and publications focused on person identification and verification using chest X-ray images, as well as other research involving Siamese networks and triplet techniques. We examine various artificial intelligence methods, including machine learning, deep learning, and attention mechanisms, highlighting specific architectures. Key factors are considered in our discussion of the latest advancements in person identification research.

Multiple studies have explored the use of chest X-ray radiographs for individual identification. Study [22] focused on utilizing chest X-ray images for person identification, moving beyond conventional biometric methods. The study highlighted the efficacy of deep learning techniques, particularly employing Siamese networks and triplet loss functions with deep pretrained models to extract features from chest X-ray radiographs, to learn and represent complex relationships within medical images. This approach aimed to improve the accuracy and reliability of person identification in the healthcare and security domains by leveraging anatomical specificity in chest X-rays. This study was the only one that used a Siamese network with three input images trained with triplet loss. This study achieved performance surpassing that of other existing state-of-the-art models, with an impressive accuracy rate of 97% in person identification using chest X-ray images. This performance surpassed that of previous studies and state-of-the-art methods, which faced challenges such as feature quality degradation and blurring. The model also demonstrated a precision of 95.3% and a recall of 98.4%, indicating its robustness in identifying individuals accurately from chest X-ray images. In the same context, [23] focused on enhancing person identification using chest X-ray images. By automatically extracting significant features from X-ray radiographs using a Siamese neural network with a VGG-16 pretrained model as a feature extractor, the proposed method aimed to improve accuracy and minimize human error in patient identification, thereby enhancing patient safety and healthcare workflows. The study demonstrated the effectiveness of the model, achieving high training accuracy (99%) and a low loss rate (0.001%).

In [24], a stochastic approach based on image processing was proposed for postmortem human identification using radiographs and CT scans. This method created a database by comparing CT scans of unidentified bodies with chest X-rays, focusing on steps such as “feature extraction”, “matching”, and “ranking”. Feature extraction derives key quantities from chest X-rays, while the matching stage compares these quantities with those stored in the database, followed by ranking similar images. The approach incorporated a decision-making process, two-level feature extraction for database and query X-rays, and similarity calculations. Initially, the bag of words (BoW) and histogram of orientations of gradients (HOG) features were extracted, with similarity scores computed in the next phase using the BoW classifier and Euclidean distance, yielding 44.4% to 63.0% accuracy. In [25], a four-step process—preprocessing, feature extraction, boundary extraction, and similarity calculation—was used to identify deceased individuals. Preprocessing enhanced contrast in radiographic images, while boundary extraction sharpened rib contours. Feature extraction applied various methods, including contrast-limited adaptive histogram equalization (CLAHE) and two-dimensional discrete Fourier transform (DFT). Euclidean distance was then used to measure similarity between feature vectors, achieving 74.07% accuracy. Study [26] focused on reidentifying individuals using deep learning and chest X-ray biometrics. Siamese neural networks (SNNs) were employed for both identification and reidentification by comparing chest radiographs to determine if they matched the same patient. Using the ChestX-ray14 dataset, the model achieved an AUC of 0.9940 and 95.55% accuracy, even identifying the same individual up to ten years later. In [27], a biometric verification method leveraged a DCNN with an EfficientNetV2-S backbone to extract features from chest X-rays. The three main steps were image acquisition, feature extraction, and identification, calculating a similarity index between clinical chest X-rays to verify patient identity. Cosine similarity measured the angle between vectors, reaching an accuracy of 83.0% on a dataset of 1000 images. This technique has potential benefits for patient safety by reducing misidentification errors. Attention mechanisms further improve accuracy by focusing on informative image features, achieving notable results across multiple applications [28]. Attention mechanisms have emerged as a powerful tool for identifying the most informative components of input images by highlighting key features and suppressing irrelevant ones, achieving significant success in various applications [28]. Recent research has focused on incorporating attention mechanisms into large-scale classification tasks. For instance, the residual attention network introduced in [29,30] employed an encoder–decoder architecture to enhance model performance in classification problems. Those studies’ work on a “Self Residual Attention Network for Deep Face Recognition” combined residual learning with self-attention to improve face recognition by using residual blocks to facilitate gradient flow and alleviate vanishing gradient issues, while self-attention modules highlighted discriminative facial regions. This model achieved state-of-the-art results on benchmark datasets such as LFW and MegaFace, boasting accuracy rates around 99.5% and 98%, respectively. The approach proposed in [30] involved a multiscale deep supervision model with attention-based feature learning designed for person reidentification (PReID). ResNet50 served as the core of the model, extracting multiple levels of deep hierarchical features from the input image. The training process involved four classification losses combined with a ranked triplet loss. To address feature information loss, a reverse attention module was integrated into the mid-level feature learning process. This module not only generated softmax scores but produces probability scores via the attention block. Additionally, a multiscale feature learning layer with deep supervision was employed during training. However, these modules were not used during testing, as they were specific to the training phase. The model demonstrated strong performance, achieving a mean average precision (mAP) of 89.0% and an accuracy of 95.5% on the Market-1501 dataset. The inclusion of the multiscale deep supervision block further enhanced the model’s performance, boosting both mAP and Rank-1 accuracy. In parallel, in [31], the authors developed the squeeze-and-excitation network (SENet), which improved classification accuracy by capturing interchannel relationships within convolutional features. Similarly, and inn [32], they introduced the convolutional block attention module (CBAM) to enhance feature selection in convolutional neural networks (CNNs). Further extending attention mechanisms, in another hand the studies [33,34] applied the framework to video classification tasks, inspired by the nonlocal means algorithm, providing a deeper understanding of self-attention models in traditional computer vision contexts. Additionally, advanced image generation processes by incorporating self-attention mechanisms to capture global dependencies within internal representations in [35]. Lastly, the authors of [36] proposed a dual attention network for scene segmentation, effectively modeling semantic interdependencies between different regions for improved segmentation accuracy.

## 3. Materials and Methods

This section focuses on the suggested method for identifying individuals using chest X-ray radiographs. We wanted to develop an accurate and dependable method for person identification and verification by leveraging the unique anatomical features present in chest X-ray images. This section provides transparency and reproducibility in our efforts to advance person identification and verification techniques by outlining the study materials, data gathering procedures, and image analysis processes.

### 3.1. Datasets

The dataset is an important part of our methodology. We trained and tested our architecture using the NIH chest X-ray [37] dataset, and an additional test of our systems using the CheXpert dataset [38].

#### 3.1.1. NIH ChestX-ray14 Dataset

The NIH ChestX-ray14 dataset, used by the researchers in [27] for evaluating the biometric identification approach therein, contains 112,120 frontal-view chest radiographs from 30,805 unique patients, making it one of the largest publicly available chest X-ray collections in the scientific community. Each patient typically has three to four images, including follow-up scans, with images provided as 8-bit PNGs of 1024 × 1024 pixels. Metadata are available for each image, detailing the patient’s age, gender, ID, and number of follow-up scans, as well as the projection view (posterior–posterior or posterior–anterior) and the presence of up to 14 common thoracic disease patterns or “no findings”. To ensure privacy, patient names were replaced with IDs, and black boxes were added over any areas with personal information in the images. This dataset was rigorously screened for personally identifiable information before release, making it suitable for public research use [37].

#### 3.1.2. CheXpert Dataset

The CheXpert dataset is a large, high-quality dataset of labeled chest X-ray images from Stanford University, widely used for training and testing medical image analysis models. It includes 224,316 chest radiographs from 65,240 patients, labeled with 14 different observations, such as pneumonia, cardiomegaly, and pleural effusion. This dataset is valuable in developing robust medical models because of its substantial size and diverse set of labels [38].

The CheXpert dataset includes a patient ID label, which is essential for tasks requiring patient-level identification and verification. This label enables tracking of individual patients across multiple images, which is exactly what our biometric system needed for testing. The patient ID label allowed us to structure the dataset for one-to-one comparisons within the Siamese model, making it possible to evaluate the model’s effectiveness in identifying and verifying unique patients based on chest X-ray images. This feature supported our system’s testing, ensuring that each embedding corresponded to a distinct patient.

### 3.2. Proposed Method

Our proposed method employed a Siamese neural network with a modified backbone architecture, trained with triplet loss, to achieve robust biometric person identification and verification using chest X-ray images. The core feature derived from our Siamese model was the embedding generator, which produced a unique 256-dimensional embedding for each input image. We used the embedding generator for extracting features from images. This section details our method, which comprised two phases, the training phase and the testing phase.

#### 3.2.1. Training Phase

**Figure 1:**
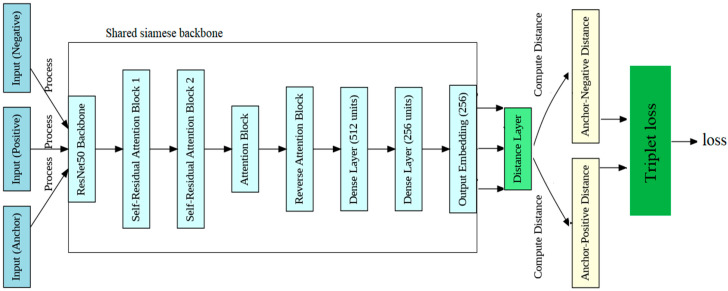
Proposed architecture.

In the training phase, the proposed method introduced a novel Siamese network and triplet architecture enhanced with self-residual attention blocks (SRABs) for person verification and identification utilizing chest X-ray images, as illustrated in Figure 1. This innovative approach leveraged the powerful capabilities of deep learning alongside attention mechanisms to tackle the complexities inherent in medical image analysis. At the heart of the proposed method lies the SRAN, which revolutionizes feature extraction by dynamically attending to relevant regions within the chest X-ray images. Through the channel attention mechanism, the network learns to emphasize critical anatomical structures and abnormalities while suppressing noise, thereby improving the model’s ability to discern subtle patterns indicative of individual identities. The spatial attention mechanism further enhances the network’s discriminative power by allowing it to adaptively focus on specific regions of interest within the X-ray images. This dynamic attention allocation enables the model to capture nuanced variations in anatomical configurations, facilitating more accurate and reliable person identification. By integrating an SRAN into a Siamese network architecture, the proposed method not only enhances feature representation but fosters robustness and interpretability. The residual connections within SRANs ensure efficient information propagation, enabling the network to effectively learn complex representations while mitigating the risk of vanishing gradients. Overall, the proposed method represents a significant advancement in medical image analysis, offering a powerful and versatile framework for person identification based on chest X-ray images. By harnessing the synergistic benefits of deep learning and attention mechanisms, this approach holds the potential to revolutionize diagnostic workflows, ultimately leading to improved patient care and clinical outcomes.

Training was conducted using triplets derived from the NIH ChestX-ray14 dataset, ensuring diverse and representative anchor–positive–negative samples. Each triplet was carefully selected from patients with at least two images to generate meaningful feature relationships. The process was carried out on Google Colab Pro+ (paid version) with a T4 GPU, and the model achieved convergence within approximately 82.7 h of training.

As shown in Figure 1, the model begins with three input images, anchor, positive, and negative, processed through the ResNet50 backbone to extract features. The output then flows into self-residual attention block 1, where feature refinement occurs through convolution, batch normalization, and Softplus activation, complemented by channel and spatial attention mechanisms that emphasize important features while using residual connections for gradient flow. The refined output is further enhanced by self-residual attention block 2, which applies similar operations for deeper feature refinement. Next, the attention block enhances the feature maps using additional attention mechanisms before passing them to the reverse attention block, which focuses on highlighting crucial features through reverse attention strategies. After flattening the enhanced feature maps, the model processes them through dense layers, yielding a final output embedding of 256 units. In order to help the model learn and optimize the triplet loss for identification and verification tasks, this embedding is then sent into the distance layer, which calculates the Euclidean distances between the anchor and both positive and negative embeddings. By making the changes mentioned above, we could reduce the information duplication across channels and identify the most crucial aspects of chest X-ray images.

##### ResNet50

ResNet50, or residual network with 50 layers, is a deep convolutional neural network architecture that helps address the vanishing gradient problem common in deep networks. It incorporates skip (or residual) connections, allowing the network to learn identity mappings, which eases the flow of information through layers without degrading performance as layers increase. ResNet50 has 50 convolutional layers, organized into bottleneck building blocks, and is known for its robust feature extraction capabilities, making it highly effective in computer vision tasks. In this work, ResNet50 serves as the head backbone of our Siamese neural network for person identification and verification, processing input images (anchor, positive, and negative) to extract initial feature embeddings. These embeddings are then refined through additional attention and residual blocks to ensure that the model focuses on identity-distinguishing features critical for accurate identification.

##### Self-Residual Attention Block (SRAB)

The self-residual attention block is a key building block that combines convolutional operations with both channel attention and spatial attention mechanisms to refine features. The input tensor X is first processed through a convolutional layer that applies a filter to extract meaningful features:

Xconv = Conv2D(X)(1)

This is followed by batch normalization and a Softplus activation to stabilize the outputs and introduce nonlinearity:

Xconv_bn = BatchNormalization(Xconv)(2)

Xconv_act = Softplus(Xconv_bn)(3)

a. Channel Attention

The channel attention mechanism helps the model focus on important channels by applying global pooling operations:

- Global average pooling (GAP):

(4) $$\mathrm{GAP}(X)=\frac{1}{H\times W}\sum_{i=1}^{H}\sum_{j=1}^{W}X(i,j)$$

- Global max pooling (GMP):

GMP(X) = max(i,j) X(i,j)(5)

These pooled features are concatenated and passed through a dense layer with a sigmoid activation:

CA(X) = σ(Dense([GAP(Xconv_act),GMP(Xconv_act)]))(6)

Finally, the attention weights are reshaped and multiplied elementwise with the input tensor to reweight the channels:

Xchannel_att = Xconv_act × CA(X)(7)

b. Spatial Attention

Spatial attention highlights important spatial regions by reducing the channel dimension:

- Average pooling across channels:

(8) $$\mathrm{AvgPool}(X)=\frac{1}{C}\sum_{k=1}^{C}X(:,:,k)$$

- Max pooling across channels:

(9) $$\mathrm{MaxPool}(X)=\underset{k}{\max}\;X(:,:,k)$$

The concatenated features are passed through a convolutional layer with a sigmoid activation:

SA(X) = σ(Conv2D([AvgPool(X),MaxPool(X)]))(10)

The spatial attention weights are multiplied elementwise with the channel-attended output:

Xspatial_att = Xchannel_att × SA(X)(11)

c. Residual Connection:

A residual connection is added to preserve gradient flow and ensure stable training. If the input tensor X has a different number of channels than the output, it is reshaped using a 1 × 1 convolution:

Xres = Conv2D(X)(12)

The final output is the sum of the spatially and channel-attended features and the residual connection:

Xout = Xspatial_att + Xres(13)

##### Attention Block

**Figure 2:**
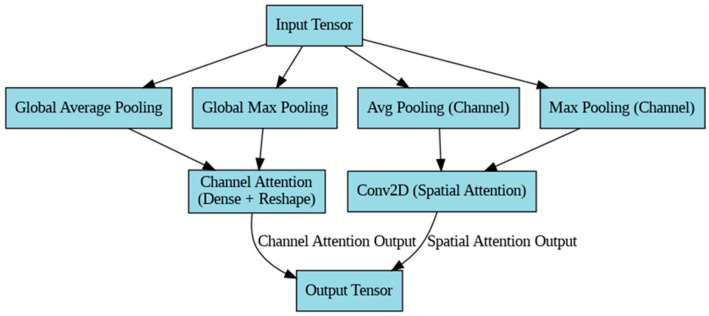
Attention block architecture.

The attention block focuses on applying attention to the already refined features using both channel and spatial attention mechanisms. It performs similar global pooling operations, but without the residual component of the previous blocks. The attention block architecture is illustrated in Figure 2

a. Channel Attention

Similarly to the SRA Block, the global average pooling and max pooling are computed:

CA(X) = σ(Dense([GAP(X),GMP(X)]))(14)

The output from the dense layer is reshaped and multiplied elementwise with the input tensor:

Xchannel_att = X × CA(X)(15)

b. Spatial Attention

The spatial attention works similarly to that in the SRA Block. Channel averages and maxima are computed and concatenated:

SA(X) = σ(Conv2D([AvgPool(X),MaxPool(X)]))(16)

The spatial attention weights are multiplied elementwise with the channel-attended output:

Xspatial_att = Xchannel_att × SA(X)(17)

Thus, the output of the attention block is:

Xout = Xspatial_att(18)

##### Reverse Attention Block

**Figure 3:**
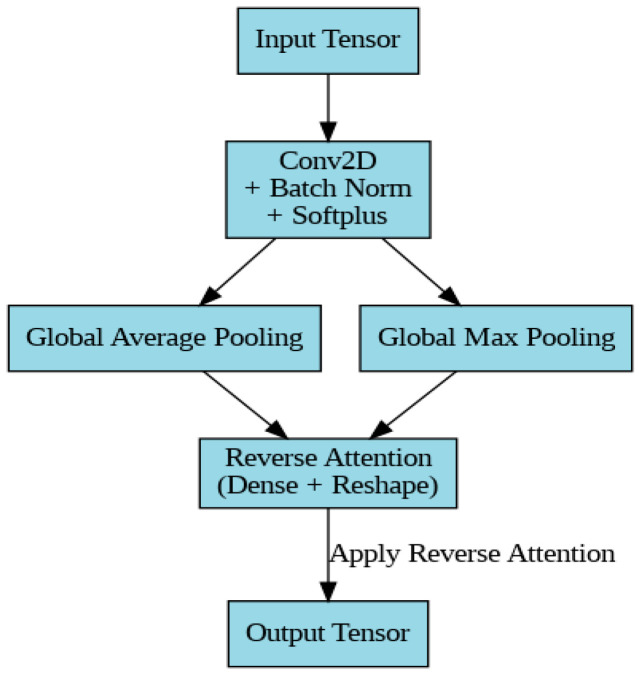
Reverse attention block architecture.

The reverse attention block is applied to reverse the attention from prior steps and highlight regions that might have been overlooked. This ensures comprehensive feature extraction. As in the previous blocks, the input tensor is first processed through a convolutional layer, batch normalization, and Softplus activation, as shown in Figure 3.

##### Reverse Channel Attention

Global average pooling and max pooling are applied similarly to before, but the reverse attention mechanism helps the model focus on regions previously downweighted:

RA(X) = σ(Dense([GAP(Xconv_act),GMP(Xconv_act)]))(19)

The reverse attention weights are then multiplied with the output:

Xreverse_att = Xconv_act×RA(X)(20)

Thus, the reverse attention block output is:

Xout = Xreverse_att(21)

##### Distance Layer

In order to calculate triplet loss, this layer calculates the Euclidean distance between the anchor and positive or negative image embeddings.

Given two vectors, A (anchor) and P (positive), the Euclidean distance between them is:

(22) $$D\_\mathrm{AP}=\sqrt{{\sum (E_{A}-E_{P})}^{2}}$$

Similarly, the Euclidean distance between the anchor and negative embedding N is:

(23) $$D\_\mathrm{AN}=\sqrt{{\sum (E_{A}-E_{N})}^{2}}$$

##### Triplet Loss

Triplet loss is a crucial training tool that ensures the model learns to differentiate between individuals by structuring the embedding space. It works by bringing similar images closer together and pushing dissimilar images farther apart. Specifically, triplet loss minimizes the distance between the anchor (A) and a positive sample (P) from the same class, while maximizing the distance between the anchor (A) and a negative sample (N) from a different class. In this setup, A represents the anchor image, P is the positive sample, N is the negative sample, and the margin hyperparameter ensures that the anchor–negative distance exceeds the anchor–positive distance by a fixed amount, reinforcing separation in the embedding space.

(24) L(A,P,N)=max(0 , D_AP−DAN+margin)

##### Data Preparation

The images were read in RGB format after being preprocessed to a consistent size of (200, 200). To guarantee that every patient had at least two samples available, we chose one image at random to serve as the anchor for each patient. Next, we randomly selected negative instances from various patients and created positive examples for each patient using the same patient ID as the anchor image. For uniformity, the RGB images were downsized to 200 × 200. Using images from the ChestX-ray14 dataset, we chose an anchor image and generated positive pairs (same patient ID) and negative pairs (different patient IDs) in order to train the Siamese network architecture using triplet loss. Additionally, we used the CheXpert dataset only for testing. We constructed actual image pairs for each subset, excluding patients who had only one radiograph, by using the patient ID labels to form positive and negative pairs.

#### 3.2.2. Testing Phase

Embedding served as the foundation for our distance-based verification and identification tasks, as we relied on the learned similarity between image embeddings to establish accurate identification. To comprehensively evaluate the effectiveness of our system, we assessed both our trained model and the same architecture without training as a baseline for comparison. The results of these analyses are detailed in the following two sections, which present identification and verification outcomes individually. We used two datasets for testing: the NIH ChestX-ray14 dataset and the CheXpert dataset. The inference time of the trained model was optimized for practical applications, with embedding generation taking approximately 3 h for 43,000 patients. The subsequent identification process required 9 min, while the verification process completed in just 1.6 min, demonstrating the system’s efficiency and readiness for real-world deployment. For inference, a GPU, such as an NVIDIA T4 in Google Colab (Pro +), V100, or RTX 4090, is needed to accelerate the embedding generation and ensure efficient computation during the identification and verification processes. Otherwise, Google Colab (Pro + for faster GPUs) could be more efficient. At least 16 GB of RAM is required for managing large image datasets and intermediate computations. For storage, a minimum of 500 GB of storage is recommended to store the model, datasets (NIH ChestX-ray14 and CheXpert), embeddings, and temporary files generated during inference. Our strong point is that we do not need to retrain our model in cases of new datasets or patients; we generate only embeddings and store them using our embedding generator.

##### Identification

**Figure 4:**
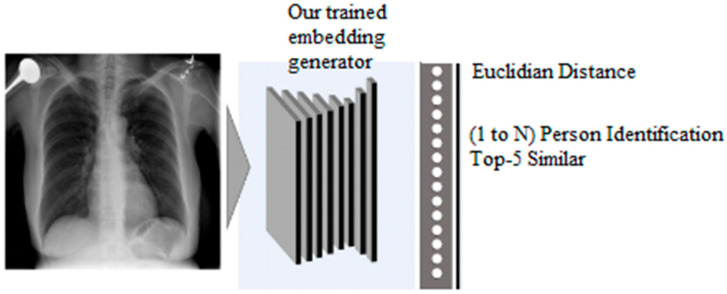
Identification system.

**Figure 5:**
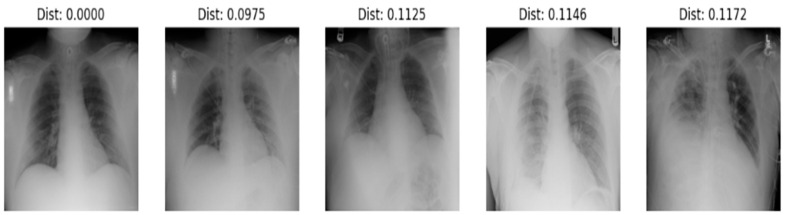
Example of testing a sample for identification.

In the identification process, shown in Figure 4 the system processes an anchor image (a reference chest X-ray) along with multiple comparison images by passing each image through the embedding generator. The model extracts a 256-dimensional embedding for each image, capturing identity-specific features. To determine identity, the Euclidean distance between the embedding of the anchor image and each comparison image is calculated. The system then ranks the comparison images by distance and selects the top 5 closest matches showed in Figure 5 example of identifying a person. This top-5 ranking approach enhances identification accuracy by allowing for slight variations between images while prioritizing those with highly similar embeddings. To integrate new patients into the system, first, embeddings are generated for each radiograph of the new patients using the proposed model. We stored these embeddings in a secure database alongside patient identifiers. When a new query radiograph is processed by the model, a fresh embedding is generated and matched against stored embeddings using our distance-based approach. The top-5 similarity search method helps to identify the patient by retrieving the closest matches. We evaluated the performance of this identification process using the nontrainable architecture to compare the effectiveness of our trained model against the baseline, as shown in Table 1.

##### Verification

**Figure 6:**
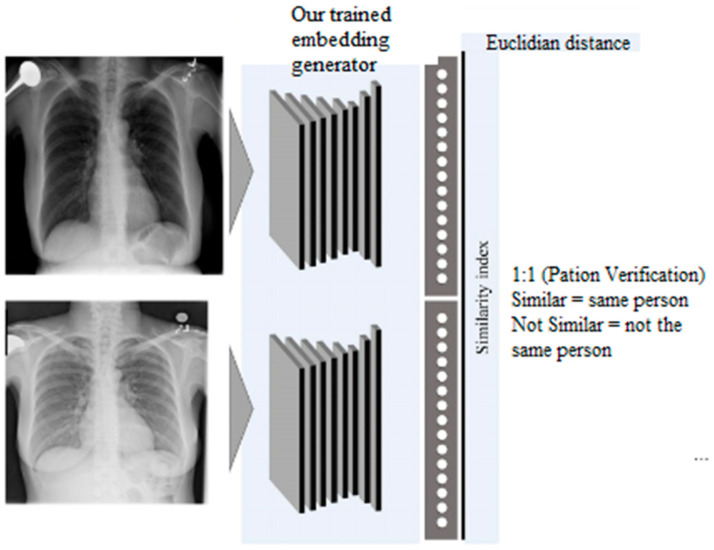
Verification System.

In the verification process, the system assesses whether two given images correspond to the same individual. Both images are passed through the embedding generator of the Siamese model, which extracts their 256-dimensional embeddings. The Euclidean distance between these two embeddings is calculated. If the distance is small (below the specified threshold), the system confirms that the two images represent the same person. Conversely, if the distance is large, the images are classified as belonging to different individuals. The proposed patient verification system, which operates on a one-to-one basis, is illustrated in Figure 6. We evaluated this verification process using the nontrainable architecture to establish a baseline for comparison against our trained model, as shown in Table 1.

## 4. Results and Discussion

**Figure 7:**
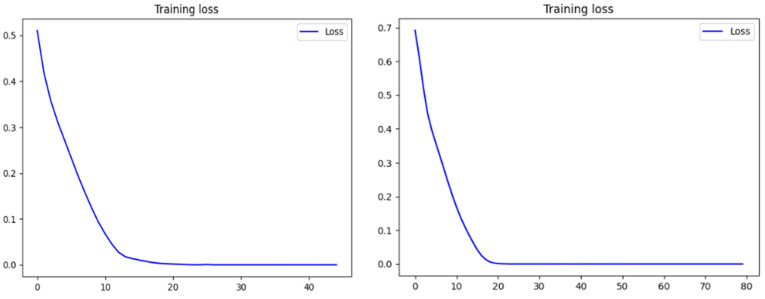
Training loss against epochs 40 and 80.

In this section, we discuss our evaluation of our trained Siamese model through multiple analyses, including the illustration of loss over 40 and 80 epochs (Figure 7), which proved that loss was stable at 0.000. Additionally, we assessed the model’s ability to generate embeddings effectively and examined its verification and identification performance. For a comprehensive comparison, we found that ResNet50, as a standalone backbone, typically trains 20–25% faster because of its reduced architectural complexity. Our previous study, referenced as [22], demonstrated an accuracy of 93% using only a pretrained ResNet50 model, and this study used other pretrained models. The ResNet50 complexity and training runtime were lower than those of our SRAN model by approximately 20–25%. Another study, referenced as [39], utilized a pretrained ResNet50 model with spatial attention, achieving 96% accuracy and a loss of up to 0.06. In contrast, our SRAN model significantly outperformed these, achieving 98% accuracy with a negligible loss of 0.0000. We also tested the model architecture without training to observe its baseline performance. The findings can be compared with the existing literature and theoretical frameworks to highlight similarities, differences, or new insights. This section reviews the empirical data obtained from the previously described experimental protocols. The primary goal is to present the results objectively, often utilizing tables, figures, and statistical analyses for clarity. By contrasting the results with prior research or theoretical expectations, we aim to uncover patterns, discrepancies, or novel discoveries. Our proposed method was implemented using the Keras library in Python (version 3.12.6). We tested our embedding generator in verification and identification using the NIH ChestX-ray14 and CheXpert datasets; the results are shown in Table 1.

The task of person identification using chest X-ray images is inherently challenging because of factors such as image quality, variations in patient anatomy, and the subtlety of distinguishing features. Existing studies have employed various methods, each with its strengths and weaknesses, illustrating the evolution of techniques in this domain, as shown in Table 2.

Early research, such as that in [24,25], utilized conventional machine learning techniques, including the histogram of oriented gradients (HOG) and two-dimensional discrete Fourier transform (DFT). While these methods represent significant initial efforts, they achieved limited success, with accuracies ranging from 44.4% to 74.07%. The challenges faced by these approaches primarily stemmed from their reliance on handcrafted features, which often struggle to capture the complex information present in chest X-ray images. Additionally, issues such as image blurring can degrade feature quality, leading to poor model performance. Study [26] introduced a Siamese neural network utilizing contrastive loss, achieving an accuracy of 95.55% on the ChestX-ray8 dataset. This method marked a notable improvement over traditional techniques, leveraging the power of deep learning to automatically extract features. However, the reliance on only positive pairs during training may have limited the model’s ability to discern subtle differences, particularly in imbalanced datasets where negative samples are more prevalent. Recognizing the limitations of contrastive loss, Studies [21,22] employed a Siamese network with triplet loss. This approach incorporated not only positive but negative samples, enhancing the model’s capacity to learn discriminative features. The architecture encouraged the network to develop a more robust feature space by promoting larger interclass separations and smaller intraclass variations. As a result, those studies achieved impressive accuracies of 97% and 96% on the ChestX-ray14 dataset, surpassing previous models. Other studies, such as those involving facial recognition [42,43], have also adopted Siamese networks and triplet loss strategies, but they have not addressed the unique challenges posed by chest X-ray images. The performance metrics from these facial recognition methods do not directly translate to our context, highlighting the distinct complexities in identifying individuals through medical imaging. Our proposed methodology strategically integrates triplet loss with self-residual attention mechanisms, allowing for a deeper and more nuanced understanding of the feature space. By effectively managing the imbalance in training data and incorporating multiple input types (anchor, positive, and negative), our model demonstrated remarkable robustness and generalizability. Our results revealed an impressive identification accuracy of 98% on the ChestX-ray14 and CheXpert databases, surpassing the performance of existing methods in the literature. The incorporation of the triplet loss function allowed our model to learn more discriminative feature representations by considering both positive and negative samples, enhancing its ability to distinguish between individuals effectively. In the landscape of chest-X-ray-based person identification and verification, prior works have laid a foundation but often fallen short in several areas that are critical for high-accuracy biometric applications. Many studies have implemented conventional convolutional neural networks (CNNs) or straightforward Siamese architectures, which, while functional, offer limited depth and adaptability. These simpler models extract only basic features, which restricts their ability to capture the complex anatomical variations present in chest X-ray images, ultimately impacting their identification performance. For instance, models utilizing shallow CNNs are typically efficient but struggle to achieve high accuracy in differentiating among individuals with similar anatomical features, as they lack both the depth and refinement that a more sophisticated architecture provides. Another common limitation in previous approaches is the absence of advanced attention mechanisms. Most prior models have applied uniform weighting to all features, meaning that they failed to focus selectively on identity-specific regions within chest X-rays. This generalized attention approach has made it difficult for these models to consistently recognize distinguishing features unique to each individual. Furthermore, previous works have rarely incorporated mechanisms to handle errors in feature extraction or to adjust for nuances in image data. Without such corrective layers, these architectures are more prone to overfitting or failing in scenarios where complex intraclass variations arise. As a result, the embedding spaces produced by these models have often suffered from high overlap between identities, which would limit their effectiveness in high-stakes identification scenarios. Optimization strategies in prior works have also differed significantly from our approach. Many of these models have used binary or categorical losses, which can be useful for basic classification but lack the granularity required for fine-grained person identification tasks. These losses fail to create the distinct separations between positive and negative embeddings necessary for robust identification, as they do not drive the anchor–positive pairs close enough in the feature space or push the anchor–negative pairs far enough apart. Consequently, models relying on these simpler loss functions are more prone to ambiguous embeddings, which can lead to lower identification accuracy. In comparison, our architecture addressed these limitations through a ResNet50 backbone for deeper feature extraction, combined with self-residual and reverse attention Blocks to focus on and refine the most distinguishing features. Furthermore, we utilized triplet loss, which directly optimizes the feature space for clear separation of positive and negative pairs, enhancing our model’s ability to differentiate individuals effectively. This approach underscored our architecture’s superior adaptability and robustness, setting it apart as a leading solution for chest X-ray-based biometric identification. Our approach also addressed a critical limitation found in previous models that relied on traditional classifiers for person identification. These classifiers, while effective in standard classification tasks, require multiple examples of each patient to be included in the training data to achieve acceptable performance levels. This constraint is particularly restrictive in biometric identification, where only a single image may be available for certain individuals. Additionally, these classifiers demand retraining each time a new individual is added to the system, making them impractical for real-world applications where patient databases are frequently updated. This need for constant retraining highlights the lack of scalability and adaptability in classifier-based approaches for large, evolving datasets. In contrast, our model’s use of distance-based metrics, such as Euclidean distance, overcomes these limitations by leveraging feature embeddings to measure similarity directly without needing repeated training. Distance metrics allow for efficient nearest-neighbor searches that scale seamlessly as new individuals are added to the dataset, enabling on-the-fly identification without the need for retraining. This approach is not only more flexible but inherently adaptable, as it evaluates each embedding based on its proximity to others rather than a fixed classification scheme. By focusing on embedding-based distance metrics, our system avoids the constraints of traditional classifiers, showcasing a more powerful and efficient solution for scalable biometric identification. Our system demonstrated remarkable improvements in both accuracy and robustness. As seen in Table 1, our trainable model achieved a substantial accuracy boost over similar architectures. Specifically, in identification tasks, our model attained 98% accuracy on the NIH dataset and 96% on the CheXpert dataset, far surpassing the 66% accuracy achieved by the same architecture without our enhancements. In verification tasks, the accuracy of our model reached 91% on the NIH dataset and 90% on the CheXpert dataset, again significantly outperforming the baseline architecture, which showed only 59% and 57% accuracy, respectively. This difference highlighted the power of our architecture’s ResNet50 backbone and sophisticated attention mechanisms, such as the self-residual, channel, and reverse attention blocks, which focus on identity-specific features in the chest X-ray images. The result is a much more discriminative embedding space, essential for biometric applications where individuals must be reliably differentiated. The enhanced performance on two prominent datasets underscored our architecture’s generalizability and reinforced its robustness across varying patient populations and imaging conditions. Table 1 illustrates how our system, with its advanced optimization and attention-driven refinements, consistently outperformed simpler architectures, marking it as a robust solution for accurate chest X-ray-based person identification and verification.

## 5. Conclusions

In conclusion, our research presents a robust and adaptable biometric identification system that advances the field of chest X-ray-based person identification and verification. By integrating a ResNet50 backbone with self-residual, channel, and reverse attention blocks, along with the application of triplet loss, our architecture demonstrated significant improvements in accuracy and feature discrimination. Unlike traditional approaches that rely on handcrafted features or simple classifiers requiring multiple images per individual, our model leveraged distance-based metrics to achieve flexible, scalable, and highly accurate identification, attaining 98% identification accuracy on the NIH dataset and 96% on CheXpert, with consistently strong verification rates. This architecture not only addresses key limitations in previous works, such as data imbalance and retraining needs, but provides a generalized solution adaptable to evolving biometric databases. Our results underscore the potential of this system to meet the demands of real-world medical imaging and biometric applications, marking a meaningful contribution to accurate, scalable, and reliable person identification using chest X-rays. For the 2% misclassification, we think that a subset of these errors was associated with some images showing signs of lower quality, such as poor resolution or positioning inconsistencies, likely due to imaging conditions that deviated from the standard protocols the model was trained on. We plan to include a detailed examination of these cases in future research. In addition to the contributions made by our research, several future directions and perspectives could further enhance the robustness and applicability of chest-X-ray-based biometric identification systems. First, integrating additional modalities, such as combining chest X-rays with CT or MRI scans, could improve accuracy by providing complementary anatomical information. Our results demonstrated the model’s robustness even without synthetic data enhancement. However, in future work, we plan to incorporate advanced data augmentation methods, such as generative adversarial networks (GANs), to synthesize high-quality, realistic X-ray images. This approach would enable us to expand the dataset effectively and create positive samples for patients who have only one image available, further improving the model’s generalization and performance. We acknowledge that the reliance on preexisting medical records for chest-X-ray-based person identification may limit its applicability, particularly in regions with limited healthcare infrastructure. However, it is worth noting that chest X-rays are one of the most widely available imaging modalities worldwide, even more so than other biometric modalities such as DNA, face images, and fingerprints. This makes chest X-rays a very important modality in the biometric field.

## Figures and Tables

**Table 1:** Performance of our system.

Model	Task	Accuracy (NIH Dataset)	Accuracy (CheXpert Dataset)
	Identification	98.3	96.1
Our trainable model	Verification	91	90.8
	Identification	56	61.2
The same architecture without training	Verification	59	57

**Table 2:** Comparison with stat state-of-the-art works.

Work	Application	Modality Used	Method	Dataset	Accuracy
[24]	Person identification	Chest X-ray	HOG BOW classifier Euclidean distance	collected from data stored in the database of Miyazaki University School of Medicine	44.4 to 63.0
[25]	Person identification	Chest X-ray	CLAHE Two-dimensional discrete Fourier transform (DFT)	collected from data stored in the database of Miyazaki University School of Medicine	74.07
[26]	Person reidentification	Chest X-ray	Siamese NN Contrastive loss ResNet50	ChestX-ray8 dataset	95.55
[27]	Person identification	Chest X-ray	EfficientNet Cosine distance	NIH ChestX-ray14 dataset	83.0
[22]	Person identification	Chest X-ray	Siamese NN Triplet loss Transfer learning	NIH ChestX-ray14 dataset	97
[40]	Palm vein detection	Palm	Siamese network with triplet loss CED, adaptive margin-based hard negative mining Generative domain-specific features	------	------
[41]	Facial detection	Face	CNN for key point extraction KNN for classification Siamese network and triplet loss	------	------
[42]	Face recognition	Face	Triplet loss Deep Siamese network K-way face recognition network	LFW dataset	94.8
[43]	Face recognition	Face	Deep Siamese network Triplet loss	------	91
[44]	Face recognition	Face	Attention feature learning ResNet50 Triplet loss	Market-1501 dataset	95.5
[29]	Face recognition	Face	Self-residual attention N	CASIA-WebFace and MS-Celeb-1M	98.3
Our	Person identification	Chest X-ray	Siamese NN Self-residual attention N Triplet loss	ChestXray14 dataset and CheXpert	98

## Data Availability

The data presented in this study are available on request from the corresponding author.
